# Filmless versus film-based systems in radiographic examination costs: an activity-based costing method

**DOI:** 10.1186/1472-6963-11-246

**Published:** 2011-09-30

**Authors:** Hiroshi Muto, Yuji Tani, Shigemasa Suzuki, Yuki Yokooka, Tamotsu Abe, Yuji Sase, Takayoshi Terashita, Katsuhiko Ogasawara

**Affiliations:** 1 Graduate School of Health Sciences, Hokkaido University, N12W5, Kita-ku, Sapporo, 060-0812, Japan; 2 Department of Radiology, Itoh Orthopaedic Hospital, 5, S2W10, Chuou-ku, Sapporo, 060-0062, Japan; 3 Faculty of Management, Dohto University, 149, Nakanosawa, Kitahiroshima, 061-1196, Japan

## Abstract

**Background:**

Since the shift from a radiographic film-based system to that of a filmless system, the change in radiographic examination costs and costs structure have been undetermined. The activity-based costing (ABC) method measures the cost and performance of activities, resources, and cost objects. The purpose of this study is to identify the cost structure of a radiographic examination comparing a filmless system to that of a film-based system using the ABC method.

**Methods:**

We calculated the costs of radiographic examinations for both a filmless and a film-based system, and assessed the costs or cost components by simulating radiographic examinations in a health clinic. The cost objects of the radiographic examinations included lumbar (six views), knee (three views), wrist (two views), and other. Indirect costs were allocated to cost objects using the ABC method.

**Results:**

The costs of a radiographic examination using a filmless system are as follows: lumbar 2,085 yen; knee 1,599 yen; wrist 1,165 yen; and other 1,641 yen. The costs for a film-based system are: lumbar 3,407 yen; knee 2,257 yen; wrist 1,602 yen; and other 2,521 yen. The primary activities were "calling patient," "explanation of scan," "take photographs," and "aftercare" for both filmless and film-based systems. The cost of these activities cost represented 36.0% of the total cost for a filmless system and 23.6% of a film-based system.

**Conclusions:**

The costs of radiographic examinations using a filmless system and a film-based system were calculated using the ABC method. Our results provide clear evidence that the filmless system is more effective than the film-based system in providing greater value services directly to patients.

## Background

In Japanese healthcare institutions, the costs and cost structures of radiographic examinations have changed following installation of picture achieving and communication system (PACS) to improve the efficiency and quality of radiology departments operations. However, precisely estimating the cost of the examination is difficult from an efficiency viewpoint because it comprises several overheads common to various examinations (e.g., equipment expenses labor costs). While direct costs can be readily and conveniently traced to a particular examination, this is not true for indirect costs. In traditional costing systems, the ratio of costs to charges (RCC) and relative value units (RVUs), usually allocate indirect costs to individual examinations based on a measure of volume. The major management limitation of the traditional cost system is that it is not strategic; that is, it allows cross-subsidies between examinations. Therefore, the changes in the examination costs, shifting from a film-based system to a filmless system, are unclear. In addition, Japanese medical personnel generally have a poor awareness of costs, one reason why costs cannot be precisely estimated.

One particular cost accounting methodology is activity-based costing (ABC). The ABC method measures the cost and performance of activities, resources, and cost objects [1,2]. Works are classified into activities, then resources are assigned to activities, and the latter are assigned to cost objects based on their use. The ABC method recognizes the causal relationships between cost drivers and activities. The advantages of ABC versus RCC and RVUs are as follows: (1) resources consumed at treatment level are more precisely defined and reflected; and (2) resources consumed by a particular cost object are directly tracked and identified to a greater degree [1]. The disadvantages are as follows: (1) ABC is the newest of the three methods, and therefore not as well known; and (2) the calculation method is complicated because of many allocation bases. ABC has been applied to health care organizations [3-6], and several researchers have applied ABC to radiographic examinations [7-9].

To our knowledge, no study has specifically addressed the changing costs structures of radiographic examinations, resulting the shift from a film-based system to a filmless system. To provide an efficient examination as part of a medical service, it is necessary for the radiologic technologist to understand the actual costs and to apply cost management processes in a filmless system. ABC can accurately calculate to a greater degree the cost of changing resources or procedures by focusing on each activity.

The primary purpose of this study is to identify the cost structure of a radiographic examination, comparing a filmless system with that of a film-based system using the ABC method. To clarify these changing costs and cost structures within the medical service, we simulated radiographic examinations in a health clinic to simplify the flow of duties.

## Methods

### Setting and subject

Two radiologic technologists were interviewed regarding the resources and flows (radiology procedures or activities) of radiographic examinations. An orthopedic health clinic was simulated for a film-based system and a filmless system. The cost objects of the radiographic examinations were lumbar (six views), knee (three views), wrist (two views), and other. The setting was such that radiographic examinations were conducted by a radiologic technologist. The ratio of new patients to re-examined patients in the out-patients department was 6:1. The number of radiographic examinations is given in Table 1.

**Table 1:** Annual number of radiographic examinations

Cost objects	New patients	Re-examined patients
Lumbar (six views)	900	600
Knee (three views)	600	400
Wrist (two views)	600	400
Other	900	600
Total	3,000	2,000

### Costs of radiographic examinations

We calculated the costs of a radiographic examination in a filmless and a film-based system, and assessed the costs or cost components. The direct costs (film cost, film-envelope cost, and film-disposal cost) were traced to each examination.

The indirect cost were allocated to cost objects based on the ABC method. First, we extracted the resources consumed by radiographic examinations. The resources assumed the following costs depreciation costs of the equipment/system (CR system, X-ray equipment, dry film imager, viewbox (*Schaukasten*), information system (PACS, etc)); maintenance costs of the equipment/system (CR system, X-ray equipment, dry film imager, information system (PACS, etc)); labor costs (radiologic technologist, medical office personnel); and other administrative expenses (hospital administration and equipment, expenses for lighting and fuel). Labor costs were calculated by multiplying the time spent doing examination activity with the hourly rate taken from the annual salary (radiologic technologist: 6,000,000 yen; medical office personnel: 2,000,000 yen (1,000 yen/h)).

Second, we defined and classified the activities carried out during radiographic examinations. These activities were recognized as a measurable minimum unit of the consumption of resources. We then classified the activities as main (directly associated with an examination) or support activities (one that supported the examination). In addition, we classified main activities as either primary or secondary activities. Primary activities included face-to-face contact with the patient, and secondary activities supported primary activities [1]. Cost pools, where the costs were grouped together, represented a single activity--similar activities were bound together into a cost pool.

Finally, we set the resource and activity drivers, which were the allocation base. These drivers are the cause of the activity and reveal the effect of the driver. The resource drivers assigned the cost of resources to activities (cost pools) and activity drivers assigned the cost of activities to cost objects.

We also set the time spent on the activity and the machine for the CR system, X-ray equipment, dry film imager, and viewbox as the allocation base.

### Sensitivity analysis

We performed sensitivity analyses to evaluate the factors that influence the cost price. Examination costs were calculated by changing each factor ("the number of examinations," "labor costs," "depreciation costs of the equipment/system," "maintenance costs of the equipment/system," "time (increasing and decreasing by the skill of the personnel or by the disease severity in the patients)," and "other administrative expenses") by 80% or 120%.

## Results

### Examination workflow and length of time

We simplified the radiographic examination workflow as follows: (1) checking previous patient's images; (2) preparing room and equipment/system; (3) patient's positioning; (4) irradiation; (5) reading images; and (6) aftercare. For each examination, we estimated the time of radiographic examination (Tables 2 and 3).

**Table 2:** Estimation of length of examination in minutes

	Lumbar (6 views)	Knee (3 views)	Wrist (2 views)	Other
Typically				
Transportation of film/order slip	0.50	0.50	0.50	0.50
Registration of patient information	0.50	0.50	0.50	0.50
Preparing room	0.25	0.25	0.25	0.25
Calling patient	0.50	0.50	0.50	0.50
Explanation of scan	0.50	0.50	0.50	0.50
Changing clothes	2.00	2.00	0.00	1.33
Positioning	4.50	3.00	1.50	3.00
Irradiation	1.50	0.75	0.50	0.92
Reading image	6.00	3.00	2.00	3.67
Aftercare	0.50	0.50	0.50	0.50
Changing clothes	2.00	2.00	0.00	1.33
Opinion/transmission	1.50	0.75	0.50	0.92
Transportation of film/order slip	0.50	0.50	0.50	0.50
Filmless system only				
Searching/checking previous image	1.00	1.00	1.00	1.00
Film-based system only				
Preparation of previous film image	2.00	2.00	2.00	2.00
Checking previous film image	1.00	1.00	1.00	1.00
Printing film	6.00	3.00	2.00	3.67
Preparation of film envelope	0.50	0.50	0.50	0.50
Checking film image	1.50	0.75	0.50	0.92

**Table 3:** Estimation of length of activity and machine *per annum *in minutes

	Filmless system	Film-based system
CR system	30,500	30,500
X-ray equipment	43,375	43,375
Viewbox		6,875
Radiologic technologist	48,250	72,625
Medical office personnel	2,500	6,500

### Resources and resource drivers

The resources used in the radiographic examinations and the resource drivers are given in Tables 4 and 5.

**Table 4:** Resources *per annum *and resource drivers for a filmless system

	Cost (yen)	Resource driver (yen)	
Direct costs			

Total	0		

Indirect costs			
CR system	1,600,000	52.5	/minute
Depreciation of CR system	1,000,000	2000	/examination
X-ray equipment	600,000	13.8	/minute
Depreciation of X-ray equipment	1,000,000	200.0	/examination
Information system	1,000,000	50.0	/view
Depreciation of information system	500,000	100.0	/examination
Radiologic technologist	2,412,500	50.0	/minute
Medical office personnel	41,667	16.7	/minute
Administration	200,000	40.0	/examination

Total	8,354,167		

Total cost	8,354,167		

**Table 5:** Resources *per annum *and resource drivers for a film-based system

	Cost (yen)	Resource driver (yen)	
Direct costs			
Film (B5)	3,180,000	159.0	/film
Film envelope	100,000	20.0	/examination
Film disposal	600,000	30.0	/film

Total	3,880,000		

Indirect costs			
CR system	1,600,000	52.5	/minute
Depreciation of CR system	1,000,000	2000	/examination
X-ray equipment	600,000	13.8	/minute
Depreciation of X-ray equipment	1,000,000	200.0	/examination
Dry film imager	400,000	20.0	/film
Depreciation of dry film imager	300,000	60.0	/examination
Viewbox	30,000	4.4	/minute
Radiologic technologist	3,631,250	50.0	/minute
Medical office personnel	108,333	16.7	/minute
Administration	200,000	40.0	/examination

Total	8,869,583		

Total cost	12,749,583		

In the filmless system, the direct cost was 0 yen and the indirect cost was 8,354,000 yen *per annum*. In the film-based system, the direct cost was 3,880,000 yen and the indirect cost was 8,870,000 yen *per annum*. It was shown that the cost of a radiographic examination was reduced by 34.5% using a filmless system. Resource drivers were identified as "number of films (irradiation)," "number of examinations," and "activity or machine time" for each resource.

### Cost of radiographic examination and cost structure

Activity costs and examination costs are given in Tables 6 and 7. During a radiographic examination in a filmless system there are 13 main activities, 3 support activities, and 12 cost pools. In a film-based system, there are 18 main activities 2 support activities, and 17 cost pools.

**Table 6:** Cost of radiographic examination for a filmless system

Cost objects			Lumbar (6 views)	Knee (3 views)	Wrist (2 views)	Other	Total	
Indirect costs								

Activity (Cost pool)	Activity driver		Cost (yen)	Cost (yen)	Cost (yen)	Cost (yen)	Cost (yen)	Rate

Main activity								
Transportation of order slip	8.33	/examination	12,500	8,333	8,333	12,500	41,667	0.5%
Registration of patient information	51.23	/examination	76,844	51,230	51,230	76,844	256,148	3.1%
Searching/checking previous image	50.00	/examination	30,000	20,000	20,000	30,000	100,000	1.2%
Preparating room	63.83	/minute	23,937	15,958	15,958	23,937	79,791	1.0%
Calling patient	63.83	/minute	47,875	31,916	31,916	47,875	159,582	1.9%
Explanation of scan	63.83	/minute	47,875	31,916	31,916	47,875	159,582	1.9%
Changing clothes	63.83	/minute	191,499	127,666	0	127,666	446,830	5.3%
Take photographs (reading image)	116.29	/minute	1,046,627	436,095	232,584	683,215	2,398,520	28.7%
Aftercare	116.29	/minute	87,219	58,146	58,146	87,219	290,730	3.5%
Changing clothes	13.83	/minute	41,499	27,666	0	27,666	96,830	1.2%
Opinion/Transmission	24.97	/view	224,769	74,923	49,949	149,846	499,488	6.0%
Transportation of order slip	25.00	/examination	37,500	25,000	25,000	37,500	125,000	1.5%

Support activity								
Imaging administration by PACS	50.00	/view	450,000	150,000	100,000	300,000	1,000,000	12.0%
Maintenance of the equipment/system	500.00	/examination	750,000	500,000	500,000	750,000	2,500,000	29.9%
Other administration	40.00	/examination	60,000	40,000	40,000	60,000	200,000	2.4%

Indirect costs total			3,128,143	1,598,849	1,165,032	2,462,142	8,354,167	100.0%

Direct cost								

Direct costs total			0	0	0	0	0	0.0%

Total cost			3,128,143	1,598,849	1,165,032	2,462,142	8,354,167	100.0%

Cost of radiographic examination			2,085	1,599	1,165	1,641		

**Table 7:** Cost of radiographic examination for a film-based system

Cost objects			Lumbar (6 views)	Knee (3 views)	Wrist (2 views)	Other	Total	
Indirect costs								

Activity (Cost pool)	Activity driver		Cost (yen)	Cost (yen)	Cost (yen)	Cost (yen)	Cost (yen)	Rate

Main activity								
Prepare previous film image	33.33	/examination	20,000	13,333	13,333	20,000	66,667	0.5%
Transportation of film/order slip	8.33	/examination	12,500	8,333	8,333	12,500	41,667	0.3%
Registration of patient information	51.23	/examination	76,844	51,230	51,230	76,844	256,148	2.0%
Checking previous film image	54.36	/examination	32,618	21,745	21,745	32,618	108,727	0.9%
Preparing room	63.83	/minute	23,937	15,958	15,958	23,937	79,791	0.6%
Calling patient	63.83	/minute	47,875	31,916	31,916	47,875	159,582	1.3%
Explanation of scan	63.83	/minute	47,875	31,916	31,916	47,875	159,582	1.3%
Changing clothes	63.83	/minute	191,499	127,666	0	127,666	446,830	3.5%
Take photographs (reading image)	116.29	/minute	1,046,627	436,095	232,584	683,215	2,398,520	18.8%
Aftercare	116.29	/minute	87,219	58,146	58,146	87,219	290,730	2.3%
Changing clothes	13.83	/minute	41,499	27,666	0	27,666	96,830	0.8%
Opinion/Transmission	24.97	/view	224,769	74,923	49,949	149,846	499,488	3.9%
Printing film (preparation of film envelope)	68.75	/view	618,750	206,250	137,500	412,500	1,375,000	10.8%
Checking film image	13.25	/view	119,260	39,753	26,502	79,507	265,023	2.1%
Transportation of film/order slip	25.00	/examination	37,500	25,000	25,000	37,500	125,000	1.0%

Support activity								
Maintenance of the equipment/system	460.00	/examination	690,000	460,000	460,000	690,000	2,300,000	18.0%
Other administration	40.00	/examination	60,000	40,000	40,000	60,000	200,000	1.6%

Indirect costs total			3,378,772	1,669,931	1,204,113	2,616,767	8,869,583	69.6%

Direct costs								

Film (B5)	159.0	/film	1,431,000	477,000	318,000	954,000	3,180,000	24.9%
Film envelope	20.0		30,000	20,000	20,000	30,000	100,000	0.8%
Film disposal	30.0	/film	270,000	90,000	60,000	180,000	600,000	4.7%

Direct costs total			1,731,000	587,000	398,000	1,164,000	3,880,000	30.4%

Total cost			5,109,772	2,256,931	1,602,113	3,780,767	12,749,583	100.0%

Cost of radiographic examination			3,407	2,257	1,602	2,521		

The cost of various radiographic examinations using a filmless system are as follows: lumbar (6 views) 2,085 yen; knee (3 views) 1,599 yen; wrist (2 views) 1,165 yen, and other 1,641 yen. With regard to the cost structure of radiographic examinations using a filmless system, "maintenance of the equipment/system" represented 29.9% of the total cost, "take photographs (reading image)" 28.7%, and "imaging administration by PACS" 12.0%.

The examination costs for a film-based system are as follows lumbar (6 views) 3,407 yen; knee (3 views) 2,257 yen; wrist (2 views) 1,602 yen, and other 2,521 yen. The greatest cost in the cost structure was that of film costs (24.9% of the total cost), then "take photographs (reading image)" at 18.8%, "maintenance of the equipment/system" was 18.0%, and "printing film (preparation of film envelope)" was 10.8%.

The primary activities were "calling patient," "explanation of scan," "take photographs," and "aftercare" for both filmless and film-based systems. These activities cost 36.0% of the total cost for a filmless system and 23.6% for a film-based system.

### Sensitivity analysis

The result of the sensitivity analyses are given in Tables 8 and 9. "The number of examinations" was the parameter that most influenced the examination costs for both systems; however, it had a greater influence on examination costs under a filmless system than that of a film-based system. For example, lumbar examinations in a filmless system ranged from 1,844 yen (88.4%) to 2,447 yen (117.3%), whereas the costs ranged from 3,201 yen (94.0%) to 3,715 yen (109.1%) for a film-based system.

**Table 8:** Sensitivity analyses of examination costs for a filmless system

		Lumbar(yen)		Knee(yen)		List(yen)		Other(yen)	
Number of examinations	80%	2,447	117.3%	1,876	117.3%	1,384	118.8%	1,933	117.7%
	120%	1,844	88.4%	1,414	88.4%	1,019	87.5%	1,447	88.2%

Labor costs	80%	1,958	93.9%	1,501	93.9%	1,107	95.0%	1,546	94.2%
	120%	2,213	106.1%	1,697	106.1%	1,223	105.0%	1,737	105.8%

Depreciation costs of equipment	80%	1,964	94.2%	1,515	94.8%	1,118	96.0%	1,557	94.8%
	120%	2,207	105.8%	1,683	105.2%	1,212	104.0%	1,726	105.2%

Depreciation costs of system	80%	2,025	97.1%	1,569	98.1%	1,145	98.3%	1,601	97.6%
	120%	2,145	102.9%	1,629	101.9%	1,185	101.7%	1,681	102.4%

Maintenance costs of equipment	80%	1,985	95.2%	1,499	93.7%	1,065	91.4%	1,541	93.9%
	120%	2,185	104.8%	1,699	106.3%	1,265	108.6%	1,741	106.1%

Maintenance costs of system	80%	2,065	99.0%	1,579	98.7%	1,145	98.3%	1,621	98.8%
	120%	2,105	101.0%	1,619	101.3%	1,185	101.7%	1,661	101.2%

Time (skill of personnel)	80%	1,978	94.9%	1,524	95.3%	1,103	94.7%	1,559	95.0%
	120%	2,193	105.1%	1,675	104.7%	1,226	105.2%	1,724	105.0%

Time (disease severity of patients)	80%	2,006	96.2%	1,530	95.7%	1,137	97.6%	1,583	96.5%
	120%	2,165	103.8%	1,667	104.2%	1,194	102.5%	1,700	103.5%

Other administrative expenses	80%	2,077	99.6%	1,591	99.5%	1,157	99.3%	1,633	99.5%
	120%	2,093	100.4%	1,607	100.5%	1,173	100.7%	1,649	100.5%

**Table 9:** Sensitivity analyses of examination costs for a film-based system

		Lumbar(yen)		Knee(yen)		List(yen)		Other(yen)	
Number of examinations	80%	3,715	109.1%	2,503	110.9%	1,797	112.2%	2,773	110.0%
	120%	3,201	94.0%	2,093	92.7%	1,472	91.9%	2,352	93.3%

Labor costs	80%	3,203	94.0%	2,120	93.9%	1,517	94.7%	2,374	94.2%
	120%	3,610	106.0%	2,394	106.1%	1,687	105.3%	2,667	105.8%

Depreciation costs of equipment	80%	3,260	95.7%	2,160	95.7%	1,546	96.5%	2,418	96.0%
	120%	3,553	104.3%	2,354	104.3%	1,658	103.5%	2,623	104.0%

Maintenance costs of equipment	80%	3,315	97.3%	2,165	95.9%	1,510	94.3%	2,429	96.3%
	120%	3,499	102.7%	2,349	104.1%	1,694	105.7%	2,613	103.7%

Time (skill of personnel)	80%	3,320	97.5%	2,196	97.3%	1,542	96.3%	2,449	97.2%
	120%	3,531	103.7%	2,343	103.8%	1,671	104.3%	2,615	103.8%

Time (disease severity of patients)	80%	3,328	97.7%	2,188	97.0%	1,574	98.2%	2,462	97.7%
	120%	3,486	102.3%	2,325	103.0%	1,631	101.8%	2,579	102.3%

Other administrative expenses	80%	3,399	99.8%	2,249	99.6%	1,594	99.5%	2,513	99.7%
	120%	3,415	100.2%	2,265	100.4%	1,610	100.5%	2,529	100.3%

## Discussion

In this study, we calculated the costs of radiographic examinations in both a filmless system and a film-based system using the ABC method. Our results indicate that examination costs and cost structures can be expressed by identifying activity costs.

In recent times, hospital management has both emphasized and relied on knowledge regarding the cost of clinical examinations. However, estimating actual examination costs using traditional costing methods is difficult because radiographic examinations include many indirect costs, and the implementation of filmless systems has increased this trend. The ABC method can visualize the operation from a cost standpoint using costing based on relevant activities. Therefore when an operation is improved or evaluated and an equipment/system is introduced or updated, the ABC method can manage the cost of the procedure or support decision-making by clarifying the issue or estimating the improvement effect. Additionally, ABC may be able to heighten the awareness of medical personnel regarding costs by calculating accessible activity costs.

The introduction of filmless systems has resulted in various cost reductions in the use of different types of photography: the higher the number of images, the greater the reduction in costs. The rate of reduction in costs was higher for lumbar examinations (6 views) (38.78%). This result demonstrates that the reduction in film cost had the greatest impact on total cost: film cost was 24.9% of the total cost.

The rate of primary activity increased by 23.6-36.0% because of the implementation of the filmless system. In particular, the activity rate of "take photographs" increased by 18.8-27.8%. This increase in the primary activity rate indicates that the system is cost-effective; therefore such primary activities can provide greater value directly to patients.

Furthermore, the present study confirms that the number of examinations had the greatest influence on examination costs using a filmless system compared with a film-based system. If the number of examinations increased or decreased using a film-based system, the effect on the examination cost was slight because the direct cost rate (e.g., film or film envelope) is higher. Conversely, for a filmless system, the number of examinations had a much greater impact on examination cost; hence a plan to increase the number of examinations is necessary.

The present study does have its limitations. First, clinic costs need to be allocated to each department to accurately calculate examination costs. However, we were interested in the change in costs or cost structures with the introduction of the new system. Therefore, in this study, a clinic's general expenses were not considered. Second, the cost objects only included the main examinations, and operations only included examination flow. Ideally every examination should be included, and re-imaging or the time required for administrative work should also be considered. In addition, the accurate measurement of time is indispensable to determine the actual cost of examinations.

## Conclusions

The costs and cost structures of radiographic examinations using a filmless system and a film-based system were calculated using the ABC method. The cost objects were lumbar (6 views), knee (3 views), wrist (2 views), and other, with costs of 2,085, 1,599, 1,165, and 1,641 yen, respectively for a filmless system.3,407, 2,257, 1,602, and 2,521 yen, respectively, for a film-based system. Our results provide clear evidence that the filmless system is more effective than the film-based system in providing services of greater value directly to patients.

## Competing interests

The authors declare that they have no competing interests.

## Authors' contributions

HM performed the investigation. HM analyzed the data. HM wrote the manuscript. HM, YT, SS, YY, TA, YS and KO interpreted the data and contributed substantially to its revision. KO conceived the study, and participated in its design and coordination and helped to draft the manuscript. All the authors read and approved the final manuscript.

## Pre-publication history

The pre-publication history for this paper can be accessed here:

http://www.biomedcentral.com/1472-6963/11/246/prepub
